# The INDEPTH (Impact of Nuclear Domains on Gene Expression and Plant Traits) Academy: a community resource for plant science

**DOI:** 10.1093/jxb/erac005

**Published:** 2022-01-28

**Authors:** Christophe Tatout, Guillaume Mougeot, Geraint Parry, Célia Baroux, Mónica Pradillo, David Evans

**Affiliations:** 1 Université Clermont Auvergne, CNRS, INSERM, GReD Clermont-Ferrand, France; 2 GARNet, Department of Biosciences, Cardiff University, Cardiff CF10 3AX, UK; 3 Arabidopsis Events UK, 13 Mayhurst Ave, Woking GU22 8DE, UK; 4 Zürich-Basel Plant Science Center, Department for Plant and Microbial Biology, University of Zürich, Switzerland; 5 Departamento de Genética, Fisiología y Microbiología, Facultad de Ciencias Biológicas, Universidad Complutense de Madrid, Madrid 28040, Spain; 6 Department of Biological and Molecular Sciences, Faculty of Health and Life Sciences, Oxford Brookes University, Oxford OX3 0BP, UK; 7 Instituto de Agrobiotecnología del Litoral, Argentina

**Keywords:** COST Action, image repository, plants, protocols, tutorials, webinars

## Abstract

This Community Resource paper introduces the range of materials developed by the INDEPTH (Impact of Nuclear Domains on Gene Expression and Plant Traits) COST Action made available through the INDEPTH Academy. Recent rapid growth in understanding of the significance of epigenetic controls in plant and crop science has led to a need for shared, high-quality resources, standardization of protocols, and repositories for open access data. The INDEPTH Academy provides a range of masterclass tutorials, standardized protocols, and teaching webinars, together with a rapidly developing repository to support imaging and spatial analysis of the nucleus and deep learning for automated analysis. These resources were developed partly as a response to the COVID-19 pandemic, but also driven by needs and opportunities identified by the INDEPTH community of ~200 researchers in 80 laboratories from 32 countries. This community report outlines the resources produced and how they will be extended beyond the INDEPTH project, but also aims to encourage the wider community to engage with epigenetics and nuclear structure by accessing these resources.

## Teaser

We present the INDEPTH Academy, a resource for plant scientists developed by the COST Action ‘Impact of Nuclear Domains on Gene Expression and Plant Traits’.

## Introduction

Despite being sessile, plants are able to adapt effectively to their changing environment through the rapid modulation of gene expression, leading to physiological changes, growth responses, or both. Central to this process are epigenetic controls involving a growing repertoire of biochemical modifications of chromatin that serve to enhance or repress the accessibility and readout of different genomic domains. The plant epigenome is increasingly appreciated for its plasticity and contribution to plant traits (Lloyd and Lister, 2022).

Less well understood is the role played by the physical separation of the chromatin within nuclear compartments in the regulation of genome functions such as transcription, replication, repair, and recombination at meiosis. Heterochromatin and euchromatin states are the paradigm of chromatin domains initially defined cytologically as regions with differing DNA density. Nowadays, chromatin domains have been extended to DNA/histone modifications patterns and molecularly as regions showing different nucleosome densities, inter- and intrachromosomal physical interactions, together with shared chromatin modifications (see INDEPTH reviews: Dumur *et al.*, 2019; Pecinka *et al.*, 2019; Pontvianne and Liu, 2020; Santos *et al.*, 2020). Chromatin domains can also define genomic regions sharing a specific location, for instance close to the nuclear periphery or to the nucleolus, known as lamina- and nucleolus- associated domains (LADs and NADs), respectively (Pontvianne and Liu, 2020).

The advent of novel molecular profiling and microscopy imaging approaches has revealed the importance of genome folding and positioning in the 3D nuclear space into chromatin domains, and to gene regulation, in both plants and animals (Bonev and Cavalli, 2016; Doğan and Liu, 2018; Baroux, 2021). In plants particularly, understanding the 3D organizing principles of the genome and that of chromatin domains bears enormous potential for crop improvement as it provides novel routes to modulate plant responses to abiotic and biotic stress and other environmental cues (Evans *et al.*, 2020).

The ‘plant chromatin biology’ field is investigated by a relatively small community, and effective interaction between those researchers who work with model and crop species was lacking until recently. The need to develop synergies within this community was the motivation behind the INDEPTH COST Action network (Box 1).

Box 1. The COST (European Cooperation in Science and Technology https://www.cost.eu/) is a funding organization for research and innovation networks. The INDEPTH project (2017–2021) (https://www.brookes.ac.uk/indepth) shares the main support mechanisms that exist across all COST Actions. These are ‘Short Term Scientific Missions’ (STSM), ‘Training Schools’, ‘Conference Grants’, and funding to improve accessibility. INDEPTH has brought together researchers from 32 countries to foster integrative plant research aiming to decipher the inter-related regulatory processes interpreting the genome in model and crop species with particular emphasis on the role of nuclear domains in gene expression control. INDEPTH was organized in five Work Groups (WGs):

- Quantitative imaging and analysis of the plant nucleus in 3D (WG1).
- Transcriptional regulation through association of chromatin domains with nuclear compartments (WG2).
- Structure of nuclear domains and the functional output for plant traits (WG3).
- Storage, data management, and integrative analysis (WG4).
- Dissemination and training (WG5).

The INDEPTH website allows searching for specific expertise, species, and techniques within INDEPTH members (https://indepth.brookes.ac.uk/search/), and describes the work groups and their main leaders (https://indepth.brookes.ac.uk/who-we-are/). More details about INDEPTH COST Action are available in Parry *et al.* (2018).

## The INDEPTH network

INDEPTH was the culmination of >5 years of prior collaborative work between members of the International Plant Nucleus Consortium (IPNC) (Graumann *et al.*, 2013). Whereas the IPNC focused on the biological relevance of the plant nuclear envelope, the group’s scientific remit was expanded to engage with researchers who explore the role of chromatin domains in the function of the nucleus and control of gene expression.

Each of the COST support mechanisms (Box 1) is predicated upon the travel of participants across borders, so the COVID pandemic has had an enormous impact on the mechanisms that COST Action support. Very much dependent on their topic, the wider cohort of COST participants had varying success embracing online interactions, while access to meetings and research facilities has been restricted. It was in this environment that the idea of the INDEPTH Academy was developed; having the primary aim of providing online content to promote and support the activities of INDEPTH participants. In doing so, it creates a lasting legacy of relevant content to facilitate research in the new working environments.

## The INDEPTH activities

**Fig. 1.**
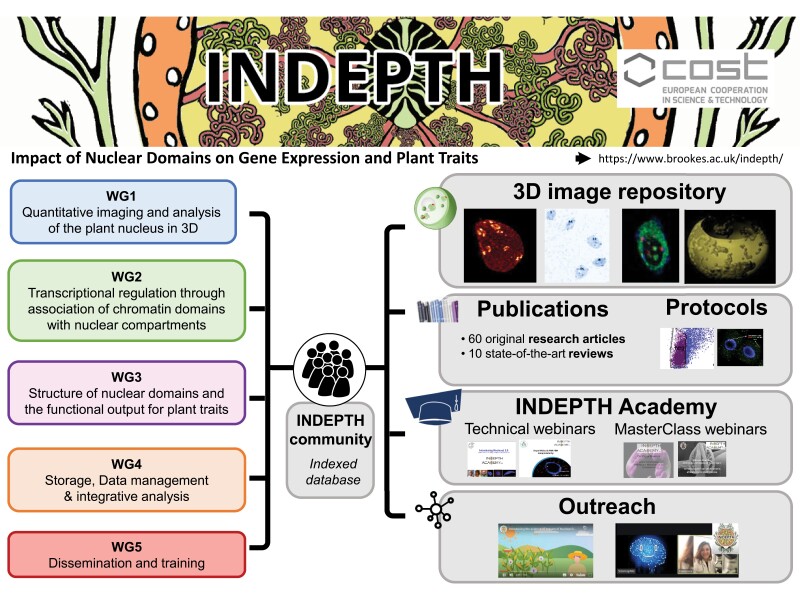
Knowledge and resources generated by INDEPTH.

INDEPTH activities were driven from the bottom-up through the activities of five work groups (WGs). Each WG represented the association of action participants whose activity, composition, and leadership were defined in order to achieve the action objectives. In this COST Action, there were four WGs (WG1–WG4) focused on different yet overlapping areas of plant chromatin biology (see Box 1 for more details and Fig. 1). In addition, WG5 was involved with Dissemination and training, of which a key element was to provide a comprehensive website (https://indepth.brookes.ac.uk/) and to link up with WG1 (Quantitative imaging and analysis of the plant nucleus in 3D) to develop an online open-access repository for 3D images of plant nuclei (https://omero.bio.fsu.edu/webclient/userdata/?experimenter=-1). This image repository provides an important community resource drawing on the strength of the network; led by Hank Bass (Florida State University, USA), the repository addressed challenges identified by the consortium and is based on the Open Microscopy Environment Remote Objects (OMERO) platform (Allan *et al.*, 2012). It is open for anyone to use, including for teaching, with user training for non-experts provided in a masterclass video by Professor Bass (https://doi.org/10.24384/cqrx-5f97). This provides an important resource for software benchmarking or to train new models for the development of deep learning (DL) tools. Efforts to automate image segmentation tailored to analyse nuclei in wide-field plant tissue images are ongoing. For instance, *NucleusJ* (Poulet *et al.*, 2015) is a plugin for *ImageJ* (Schneider *et al.*, 2012) dedicated to the characterization of nuclear morphology and chromatin organization in 3D.

## The INDEPTH Academy

The INDEPTH consortium comprises a plethora of conceptual and experimental expertise relevant for research on the biology of the nucleus and chromatin in plants. Our consortium and others (Baker, 2016) noticed that one current major challenge for research is a crisis of reproducibility, with studies showing that much published work cannot be replicated by other groups. This is a multifaceted problem, driven by poor quality and accessibility of research data, and protocols and methods that are of poor quality. The INDEPTH network has sought to address these issues through the INDEPTH Academy. The INDEPTH Academy provides a virtual community resource aimed at making this expertise widely available beyond the INDEPTH consortium, through a series of protocols, teaching seminars, and training webinars. The COVID pandemic necessitated that the INDEPTH Academy became a virtual resource, available at the INDEPTH Academy website and YouTube Channel. One important consideration of the INDEPTH Academy is that it is hosted by the RADAR (Research And Digital Assets Repository) repository at Oxford Brookes University. After uploading to the INDEPTH page at RADAR, each contribution obtains a permanent DOI, thus providing a referenceable record that can be added to CVs or similar documents.

The INDEPTH Academy includes the following. (i) INDEPTH protocols: free-to download experimental protocols that have been developed in collaboration with INDEPTH members, based on recent peer-reviewed publications. These protocols are a useful starting point for anyone new to these techniques (Table 1). (ii) Teaching webinars: the INDEPTH Academy includes a series of talks from world experts in topics related to INDEPTH activities. These are designed to be used in teaching or research as an introduction to these topics, allowing students to learn from experts in the field rather than relying on second-hand explanations from their tutors. In the future, these videos can be regularly integrated into relevant curricula. Masterclass tutorials are a key component of the INDEPTH Academy strategy to provide community resources and are provided both through the INDEPTH Academy website and on YouTube (Table 2).

**Table 1.** Standardized protocols to study the plant nucleus

Protocol	Advising author(s)	Work Group	DOI
Hi-C protocol for analysis of plant nuclear chromatin interactions	Chang Liu and Silin Zhong	WG4	https://doi.org/10.24384/39fw-jm66
Single molecular fluorescence *in situ* hybridization	Stefanie Rosa	WG2	https://doi.org/10.24384/4h9v-ar37
Localizing total mRNA in plant cells	Geraint Parry	WG2	https://doi.org/10.24384/m5dm-he87
Fluorescence-activated nuclear sorting (FANS)	Ortrun Mittelsten Scheid	WG2	https://doi.org/10.24384/fvmf-km57
Protocol for isolation of plant nucleolus	Frederic Pontvianne	WG2	https://doi.org/10.24384/ycxk-1685
Assay for transposase-accessible chromatin (ATAC-seq)	Roger Deal and Kenneth Berendzen	WG4	https://doi.org/10.24384/y5pn-8a86
Membrane yeast two-hybrid (MYTH)	Christophe Tatout and Emmanuel Vanrobays	WG2	https://doi.org/10.24384/4zmm-x149

**Table 2.** Teaching webinars on chromatin domains and chromatin dynamics in the plant nucleus

Webinar Title	Author	Work Group	Weblink
Endoreduplication in tomato fruit growth	Christian Chevalier	WG3	https://www.youtube.com/watch?v=_NKvNk_DT5Q
Linker histones	Célia Baroux	WG1	https://www.youtube.com/watch?v=pjZzUvoDDiw
Introduction to the plant nuclear pore complex	Geraint Parry	WG2	https://www.youtube.com/watch?v=ENcNVh_jK0c
The role of chromatin domains in plant meiosis	Mónica Pradillo	WG3	https://www.youtube.com/watch?v=9csbX9ScEpc
Temperature and sexual reproduction	Danny Geelen	WG3	https://www.youtube.com/watch?v=MQ5AslR8WAk
Functions of plant nucleolus	Frederic Pontvianne	WG2	https://www.youtube.com/watch?v=qIZG5qu1bcs
The fascinating biology of plant pathogenic viroids	Kriton Kalantidis	WG3	https://www.youtube.com/watch?v=JCCRdpirPrk

These teaching webinars also include short talks related to short-term scientific missions (STSMs). STSMs have been very successful during the 4 years of INDEPTH, with 31 achieved, mostly involving young researchers at the PhD or post-doctoral levels, with promise to continue their careers in plant science. STSMs offer a range of cross-sectorial and interdisciplinary training and scientific exchange activities to enhance their job opportunities. Three STSMs were selected for short webinars and included in our YouTube channel (Table 3).

**Table 3.** Teaching webinars of selected short-term scientific missions (STSMs)

STSM talk titles	Author	Work Group	Weblink
Understanding the impact of chromatin organization on transcriptional memory in plants	Emilia Cepowska	WG2	https://www.youtube.com/watch?v=H_2nFwCLbBw
Gene dosage composition of rDNA	Francesca Lopez	WG2	https://www.youtube.com/watch?v=9OtOyNQFyb4
Hi-C analysis of H1-mediated chromatin organization	Gianluca Teano	WG2	https://www.youtube.com/watch?v=fKn-dGlIj2s

(iii) Masterclass tutorials: a key element of any COST Action is to provide expert training in new experimental techniques. These short video tutorials provide an introduction about new techniques useful for chromatin and nuclear organization studies. Each tutorial is followed by a short discussion moderated by the host to broaden perspectives (Table 4).

**Table 4.** Masterclass tutorials on experimental techniques to study the plant nucleus

Masterclass Tutorials Title	Presenting expert(s)	Work Group	Weblink
Spatial analysis of nuclear signal distribution using IMARIS^Bitplane^ and DATAVIZ^UZH^	Célia Baroux	WG1	https://www.youtube.com/watch?v=tQzSOQ6RQzE
NucleusJ2.0	Christophe Tatout and Sophie Desset	WG1	https://www.youtube.com/watch?v=og4LGkLcF3w&t=48s
INDEPTH OMERO 3D Image Repository	Hank Bass	WG1	https://www.youtube.com/watch?v=t0JvvbRmHcs&t=195s
Fluorescence-activated nuclear sorting (FANS)	Ruben Gutzat	WG2	https://www.youtube.com/watch?v=K4cm7QIYdGg
Single molecular fluorescence *in situ* hybridization	Stefanie Rosa	WG2	https://www.youtube.com/watch?v=Gcl7unippao
Active segmentation as a tool for plant image analysis	Dimiter Prodanov	WG1	https://www.youtube.com/watch?v=jt2P7SQSuz4
Plant functional proteomics	Vicente Rubio	WG2	https://www.youtube.com/watch?v=9xnGuXkhimQ
Size-exclusion chromatography coupled to multiangle light scattering (SEC-MALS)—a powerful tool for characterization of macromolecular complexes	Mariusz Czarnocki-Cieciura	WG4	https://www.youtube.com/watch?v=DxhouZbgWs4

## The INDEPTH 3D image repository

Making further progress in the study of chromatin structure and organization requires microscopy imaging and a dedicated image analysis workflow to resolve the spatial distribution and topology of chromatin domains (Dumur *et al.*, 2019). With a slow start a decade ago, the plant chromatin community produces an increasing number of 3D images reporting on the nuclear distribution of chromatin and chromatin-binding components in different plant tissues and plant species. This rich source of information is, however, not easily accessible and remains dispersed among different repositories hosted by author labs or by journals. Facilitating access to these images enables scientists to harness each other’s work for comparative analyses, as is already common practice in the ‘omics’ field. Ideally as well, replicate or unused image datasets that are no longer exploited in the host lab may find a legacy and novel application in the community. A better sharing of image resources for plant chromatin and the nucleus would thus be a win–win situation for both the contributor and the operator. Another predicted benefit of such an image repository is the sharing of large image datasets for training image analysis custom software. Indeed, analysing a large number of images is necessary to capture biological variability, but the required automation is increasingly challenging as resolution and data volumes increase. To answer these demands, we created the INDEPTH OMERO image repository (Dumur *et al.*, 2019) to provide open access to >3000 3D images from plant species classified in teaching (IDP1000 series), training (IDP2000), and published (IDP3000) and unpublished (IDP4000) images (Table 5). The INDEPTH OMERO repository is hosted at Florida State University and will remain after INDEPTH ends. Images from the repository are fully accessible to anyone outside INDEPTH.

**Table 5.** List of 3D images included in the INDEPTH OMERO repository

Projects	Names	Species and samples	Staining and image pre-treatment	Microscopy parameters	OMERO links	Number
**TEACHING IMAGES**	IDP1001	*Zea mays* inbred cultivar W22 root tip cells	DAPI	Olympus 1-UB932 ×63/1.4 Oil 0.11 × 0.11 × 0.25	https://omero.bio.fsu.edu/webclient/?show=project-3603	2
	IDP1002	*Zea mays* inbred cultivar W22 root tip cells	DAPI-EDU	Olympus 1-UB932 ×63/1.4 Oil 0.07 × 0.07 × 0.20	https://omero.bio.fsu.edu/webclient/?show=project-3604	18
	IDP1003	*Zea mays* inbred cultivar W22 meiosis diakinesis stage	DAPI Deconvolution	Olympus 1-UB932 ×63/1.4 Oil 0.11 × 0.11 × 0.20	https://omero.bio.fsu.edu/webclient/?show=project-3605	7
	IDP1004	*Zea mays* inbred cultivar W22 meiosis zygotene stage	DAPI, DNA FISH FITC (telomeres), DNA FISH Rhodamine (chromosome9)	Olympus 1-UB932 ×63/1.4 Oil 0.11 × 0.11 × 0.30	https://omero.bio.fsu.edu/webclient/?show=project-3606	5
	IDP1005	*Arabidopsis thaliana* ecotype Col-0 meiosis	DAPI, Immunolocalization of ASY1, ZYP1, MLH1and RAD51 (FITC)-ASY1 (Cy3)	Olympus DP71 × 20 0.32 × 0.32×na	https://omero.bio.fsu.edu/webclient/?show=project-3607	215
**TRAINING IMAGES**	IDP2001	*Arabidopsis thaliana* ecotype Col-0, mutant *crwn1 crwn2* cotyledon	DAPI	Leica MMAF+OptiGrid ×63 water 0.10 × 0.10 × 0.20	https://omero.bio.fsu.edu/webclient/?show=project-3601	77
	IDP2002	*Arabidopsis thaliana* ecotype Col-0, mutant *kaku4 crwn1 crwn4* cotyledon	DAPI	Leica MMAF+OptiGrid ×63 water 0.10 × 0.10 × 0.20	https://omero.bio.fsu.edu/webclient/?show=project-3602	28
**PUBLISHED IMAGES**	IDP3001	*Arabidopsis thaliana* ecotype Col-0 cotyledon	DAPI	Leica MMAF+OptiGrid ×63 water 0.10 × 0.10 × 0.20	https://omero.bio.fsu.edu/webclient/?show=project-2151	77
	IDP3002	*Zea mays*, *Arabidopsis thaliana*	DAPI, immunofluorescence, and DNA FISH	Multiple	https://omero.bio.fsu.edu/webclient/?show=project-1658	30
	IDP3003	*Zea mays* inbred cultivar W22 root tip cells	DAPI-EDU	Olympus 1-UB932 ×60/1.4 0.07 × 0.07 × 0.20	https://omero.bio.fsu.edu/webclient/?show=project-2703	287
	IDP3004	*Arabidopsis thaliana* ecotype Col-0 and *3h1* mutant isolated leaf nuclei	DNA staining (580CP dye) and DNA FISH 45S rDNA+180 bp satellite repeat	Leica SP8R WL 3×STED 0.02 × 0.02 × 0.13	https://omero.bio.fsu.edu/webclient/?show=project-2351	2
	IDP3005	*Arabidopsis thaliana* ecotype Col-0 root tip cells	DAPI (cyan/blue), EdU (AF488, green) and DNA FISH 45S rDNA (AF594, red)	Zeiss Axioimager Z1 ×63/1.4 Oil 0.08 × 0.08 × 0.11	https://omero.bio.fsu.edu/webclient/?show=project-2852	11
	IDP3006	*Arabidopsis thaliana* ecotype Col-0, mutant *kaku4 crwn1 crwn4* cotyledon	DAPI DAPI -DNA FISH 5S rDNA ()- 180 bp satellite repeat	Leica MMAF+OptiGrid Zeiss SP8 × 63 water 0.10 × 0.10 × 0.20	https://omero.bio.fsu.edu/webclient/?show=project-2801	2477
	IDP3007	*Arabidopsis thaliana* ecotype Col-0 and *3h1* mutant isolated leaf nuclei	DNA staining= 580CP dye Deconvolution	Leica SP8R WL 3×STED 0.04 × 0.04 × 0.06	https://omero.bio.fsu.edu/webclient/?show=project-2201	6
**UNPUBLISHED IMAGES**	IDP4001	*Arabidopsis thaliana* ecotype Col-0 cotyledon	DAPI	Leica MMAF+OptiGrid ×63 water 0.10 × 0.10 × 0.20	https://omero.bio.fsu.edu/webclient/?show=project-2551	10
	IDP4002	*Arabidopsis thaliana* ecotype Col-0 cotyledon	DAPI	Leica MMAF+OptiGrid ×63 water 0.10 × 0.10 × 0.20	https://omero.bio.fsu.edu/webclient/?show=project-3451	413

**Fig. 2.**
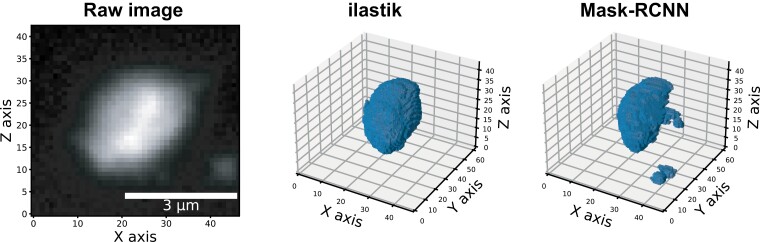
Image segmentation of a 3D nucleus from the IDP4002 dataset through the Mask-RCNN deep-learning model. Example of a raw image of a 3D nucleus from the IDP4002 dataset (left), the corresponding 3D segmentation performed semi-automatically with ilastik (middle), and the automatic segmentation obtained with Mask-RCNN pre-trained on a 2D dataset of nuclei (right). The small extra objects segmented in this last image illustrate the noise sensitivity of Mask-RCNN on our dataset and the need for retraining it.

As an example of use of this resource, Dubos *et al.* (2020) uploaded all their raw and segmented images to make them available to the community. Our datasets are useful for software benchmarking (Bilalovic *et al.*, 2020; Dubos *et al.*, 2021, Preprint) and to train future DL-based algorithms as discussed by Dimiter Prodanov (Interuniversity Microelectronics Centre-IMEC, Belgium) and Sumit Vohra (Zuse Institute, Germany) during one of the INDEPTH Academy Masterclasses (Active Segmentation Tool for plant image analysis, www.youtube.com/watch?v=jt2P7SQSuz4). To exemplify this, we initiated the preliminary steps toward the benchmarking of DL methods dedicated to image segmentation. Many DL methods have recently been made available and are promising tools to achieve nuclear segmentation from microscopy images (Moen *et al.*, 2019). The basic principle to define the performance of a given DL method relies on comparing the output of the model with the expected results called the ground truth segmentation, that is usually a manually or semi-automated segmentation of a large image dataset. To this aim, we generated a new ground truth dataset called (IDP4002, *n*=413 nuclei) using Ilastik (Berg *et al.*, 2019) that is made of segmented images obtained from a semi-automated annotation of raw images of nuclei from IDP3006. The ground truth dataset was then used to explore a first DL model called Mask-RCNN that is one of the most popular tools in image segmentation (He *et al.*, 2020). As a first try, we pre-trained Mask-RCNN on the 2D nucleus using a training routine provided by the developers of Mask-RCNN (He *et al.*, 2020) (https://github.com/matterport/Mask_RCNN) and the Data Science Bowl 2018 dataset (Caicedo *et al.*, 2019). The pre-trained model was then used to segment our raw dataset in a slice-by-slice process in order to reconstitute a 3D segmentation (Fig. 2). The performance of the prediction was then evaluated using the Dice and Jaccard indices by comparing the expected results (i.e. the ground truth segmentation) with the Mask-RCNN output. The performance of this first trained model ranged from 0.380 (Jaccard index) to 0.504 (Dice index), meaning that the pre-trained model was not well adapted to 3D segmentation and will need further adjustment. Alternatively, more recent DL methods dedicated to the 3D nucleus such as Cellpose (Stringer *et al.*, 2021) or Stardist (Weigert *et al*., 2020) could be compared using the IDP4002 dataset. The dataset size will let us both train these methods and evaluate them on plant nuclei as well as allowing us to create new and more efficient methods.

## The INDEPTH legacy

Over the past 4 years, the INDEPTH network has facilitated the development of many research projects that have resulted in high-profile peer-reviewed publications. These include, among many others, exploring the function of Arabidopsis rRNA genes (Lopez *et al.*, 2021), developing an understanding of why mutants of the SMC5/6 complex often develop abnormal seeds and genome instability in tetraploid conditions (Yang *et al*., 2021*a*, *b*), and determining how polycomb-dependent chromatin compartmentalization influences gene transcription (Huang *et al.*, 2021). INDEPTH members edited a Special Issue of the *Journal of Experimental Botany* named ‘Impact of Chromatin Domains on Plant Phenotypes’. This Special Issue brought together 13 reviews and one original research article on this topic and will remain an important reference issue for the next decade. In addition, INDEPTH members have been involved in recent special issues with the journals *Frontiers in Plant Science* and *Nucleus*, and promoted the establishment of INDEPTH with a high-profile meeting report (Parry *et al.*, 2018). Therefore, it is important that the excellent community efforts of the INDEPTH group are maintained through an appropriate long-term forum.

INDEPTH Academy content will continue to grow in the coming period with new webinars and protocols. The legacy of INDEPTH is now established within the Cell Section of the Society of Experimental Biology (SEB). A recently formed SEB Special Interest Group (SIG) with a focus on nuclear dynamics and plant reproduction will secure the legacy of INDEPTH collaboration through regular participation in SEB meetings and representation in the SEB, meaning that the society will be the future home of the large pan-European INDEPTH community. The INDEPTH Academy provides a unique starting resource for scientists studying the functional organization of plant genomes in the nucleus with the goal of elucidating how chromatin domains influence gene expression and ultimately plant traits. The legacy of this resource also depends on the active contribution of its community, which we are actively encouraging. Together with resources provided by related networks, such as the International Nucleome Consortium (INC, https://inc-cost.eu/) and Epigenetic Mechanisms of Crop Adaptation to Climate Change (EPI-CATCH, https://www.epicatch.eu/), the INDEPTH Academy will hopefully contribute sustainable and collective efforts to bring together knowledge and data sharing.

The INDEPTH website and its resources are secured, and plans are in place to integrate INDEPTH Academy resources into the SEB website, to continue to implement these repositories, and to increase the visibility of this important plant science community. This integration has already begun; INDEPTH collaborated with the SEB to organize INDEPTH Academy talks as part of the 2021 SEB conference session ‘Dynamic organization of the nucleus across kingdoms’. From this, the SEB produced a YouTube resource ‘INDEPTH sessions—SEB Conference 2021’ (https://www.youtube.com/playlist?list=PLbQMipJZx271UHDa2cfofMDdDeDL6lUW9) making available 14 webinars from INDEPTH members, which will complement INDEPTH Academy talks from this event at which members of the community introduce their current research.

## Conclusion

INDEPTH has successfully gathered a large community to collaborate and exchange knowledge and techniques in the exciting and new field of plant nuclear domains. In order to foster synergies in plant nuclear domain research in plants, INDEPTH has created INDEPTH Academy, which includes multimedia resources such as webinars and interactive discussions about how the spatial organization of nuclear DNA is critical in the adaptation to environmental stresses. INDEPTH Academy, developed as a response to the situation created by the COVID-19 pandemic in the context of the COST Action ‘Impact of Nuclear Domains on Gene Expression and Plant Traits’, provides a basis for sharing expertise and developing new techniques in this field.

## Abbreviation

COST: European Cooperation in Science and Technology
COVID: coronavirus disease
DL: deep learning
INDEPTH: Impact of Nuclear Domains on Gene Expression and Plant Traits
IPNC: International Plant Nucleus Consortium
OMERO: Open Microscopy Environment Remote Objects
SEB: Society of Experimental Biology
STSM: short-term scientific mission
WG: work group
